# Activity artifacts in drug discovery and different facets of compound promiscuity

**DOI:** 10.12688/f1000research.5426.1

**Published:** 2014-10-03

**Authors:** Jürgen Bajorath

**Affiliations:** 1 Department of Life Science Informatics, B-IT, LIMES Program Unit Chemical Biology and Medicinal Chemistry, Rheinische Friedrich-Wilhelms-Universität, Bonn, D-53113, Germany

## Abstract

Compounds with apparent activity in a variety of assays might disable target proteins or produce false assay signals in the absence of specific interactions. In some instances, such effects are easy to detect, in others they are not. Observed promiscuity of compounds might be due to such non-specific assay artifacts. By contrast, promiscuity might also result from specific interactions with multiple targets. In the latter case, promiscuous compounds can be attractive candidates for certain therapeutic applications. However, compounds with artificial activity readouts are often not recognized and are further progressed, which presents a substantial problem for drug discovery. In this context, the concept of PAINS (pan-assay interference compounds) should be seriously considered, which makes it possible to eliminate flawed compounds from the discovery pipeline, even if their activities appear to be sound at a first glance.

## Correspondence

In a recent commentary, Baell and Walters specify the threat to drug discovery programs that comes along with PAINS (pan-assay interference compounds) ^1^. PAINS are small molecules that fake biological (target-specific) activities in assays, by chemically disabling target proteins or producing false assay signals (e.g., through color effects). PAINS do their fatal job through a variety of unwanted chemical mechanisms including, among others, covalent modifications, chelation of metal ions essential for catalytic functions redox effects or by disrupting membrane environments required for receptor integrity. The PAINS concept was originally introduced Baell and Holloway ^2^ and might also be viewed in context of earlier work on frequent hitters in screening assays by the Shoichet group ^3^. Frequent hitters cause non-specific protein aggregation, micelle formation, or denaturing effects and thereby also produce activity artifacts. The PAINS concept is knowledge-based and it is evident that a high level of chemical expertise and much careful research have been prerequisites for its introduction and further refinement.

In their off-the-beaten-path contribution, Baell and Walters detail PAINS liabilities. They provide evidence that PAINS often make it into the discovery pipeline and that their destructive deeds might be discovered late in the game, if at all, thereby wasting valuable resources. As leading experts, the authors do not hesitate to admit that they themselves have made PAIN(S)ful experiences in the past that have inspired them to dig deep and get to the roots of the problem. Indeed, PAINS often progress below the radar screen of chemical awareness and are difficult to detect when observing apparent (yet artificial) dose-response behavior and/or pseudo-SARs (structure-activity relationships).

Baell and Walters point out that they have identified about 400 compound classes (!) representing PAINS, but that consideration of only 16 major classes is sufficient to eliminate more than half of PAINS present in screening libraries. Among others, these primary suspects include hydrogen peroxide producing molecules such as toxoflavins, covalent modifiers such as isothiazolones and rhodanines, or compounds whose degradation products might produce artificial signals in many assays such as phenol-sulfonamides. Baell and Walters provide guidance on how to best identify PAINS early on and prevent the progression of flawed compounds, for example, through the use of orthogonal assays to re-evaluate screening hits or computational (substructure) filters to detect PAINS. The latter approach is easy to implement and can be used on a routine basis to screen compound collections for major classes of PAINS.

Interestingly, Baell and Walters attribute PAINS progression in discovery projects primarily to the naivety of medicinal chemists or drug discovery researchers in academia, a point that might be perceived as controversial by many. After all, potential PAINS pitfalls of academic scientists are easier to spot than those of their colleagues in the pharmaceutical industry because academic accidents are primarily manifested in publications, whereas failures in pharma environments are typically not publicized. On a lighter note, the discussion of Baell and Walters is supported by truly ‘innovative’ display items that should help to open up (even ugly) chemistry to the masses.

From reviewer and editorial experiences, one can attest to the fact that PAINS present a problem for the scientific literature and often go unnoticed. For example, the *Journal of Medicinal Chemistry* frequently receives submissions reporting hits from experimental or computational (virtual) screening campaigns with PAINS liability (and is currently taking appropriate measures to tackle these problems, in collaboration with Jonathan Baell). Hence, there are all good reasons to raise the awareness of these issues and provide catalogues of PAINS as references for investigators in academia and the pharmaceutical industry. Of course, it is not certain that each and every compound containing a PAINS (sub)structure will be a ‘chemical con artist’ (to use Baell’s and Walters’ terminology), as compound reactivity or other effects might also be context-dependent. Any PAINS alert, however, should trigger careful follow-up studies to re-evaluate activity readouts.

In the context of PAINS and frequent hitters, another aspect should also be carefully considered, i.e., the Janus headed issue of compound promiscuity ^4^, which is often misunderstood. Promiscuity might well be associated with non-specific effects. Compounds active in many different assays are indeed likely to represent PAINS or frequent hitters due to the artifacts discussed above. This type of compound promiscuity might be best rationalized as ‘assay promiscuity’. By contrast, promiscuity also results from the ability of small molecules to specifically interact with multiple targets ^4^, and this type of ‘target promiscuity’ provides the molecular basis of ‘polypharmacology’ ^5^. In certain therapeutic areas such as oncology, the efficacy of a drug often depends on its ability to specifically bind to multiple targets and elicit polypharmacological effects (i.e., interference with multiple signaling pathways), with kinase inhibitors being a prime example.

Large-scale mining of assay data has revealed different facets of promiscuity ^4, 6^. Analysis of compounds from 1085 confirmatory bioassays for 439 targets available in PubChem ^7^ has shown that a screening hit interacted with, on average, two targets, provided that only high-confidence activity data were considered ^6^. This reflects a fairly low level of target promiscuity, although nearly 80% of all active PubChem compounds were tested in more than 50 different assays. The probability of an active compound to interact with at least two targets was calculated as ∼50% and the probability of interacting with more than five targets was less than 8%. However, even under the most stringent activity data selection criteria, more than 2000 hits (∼0.45% of PubChem’s confirmatory bioassay compound collection) were detected that were active against more than 10 targets (consistently displaying dose-response behavior) ^6^. In these cases, boundaries between target and assay promiscuity become rather fluid, and promiscuous compounds and their activities should be further investigated. Moreover, 160 compounds displayed activities against more than 20 targets and many of these highly promiscuous PubChem compounds were PAINS, according to Baell *et al*.

Compounds that are not PAINS or frequent hitters might occasionally also display high levels of assay promiscuity. For example, under the conditions of small molecule microarray experiments, some compounds from diversity-oriented synthesis and other sources were found to be active against more than 90 (!) sequence-unrelated targets ^8^. Small chemical modifications of these highly promiscuous molecules (identified through matched pair analysis of library compounds) often dramatically reduced their microarray activities or rendered them completely inactive ^8^. Hence, for such structural analogs, assay and target promiscuity would be very difficult to distinguish in the context of a given experiment, which would require follow-up assays under different conditions.

The PAINS concept put forward by Baell *et al.* is a milestone event for medicinal chemistry and drug discovery, just as the first detection of frequent hitters by McGovern *et al.* has been more than a decade ago ^3^. Without doubt, focusing on flawed compounds represents a major obstacle for drug discovery research, be it in academia or the pharmaceutical industry, and so do publications reporting, in good faith, apparent activities of such compounds. Care must also be taken to distinguish between (true) target and assay promiscuity of active compounds and be aware of experimental situations where this might not be possible.

It has taken a fairly long time until the concept of frequent hitters was generally accepted in biological screening and it will take time until there is general awareness and routine consideration of PAINS in the practice of medicinal chemistry and drug discovery. To these ends, the commentary of Baell and Walters makes an invaluable contribution. To a wider audience (hopefully including many students) it also demonstrates that serious problems in chemistry can be dealt with in an equally thought-provoking and entertaining manner.
