# Supplementary material for: Activity artifacts in drug discovery and different facets of compound promiscuity
Source: F1000Res. 2014 Oct 3;3:233. [Version 1] doi: 10.12688/f1000research.5426.1 (PMC4193391; doi:10.12688/f1000research.5426.1)
Supplement: Peer review history

# Peer review: Referee response for version 1

**DOI:** 10.5256/f1000research.5795.r6319

**Published:** 2014-10-09

**Authors:** Bill Greenlee

**Affiliations:** 1 MedChem Discovery Consulting LLC, Teaneck, USA

This is an interesting and informative paper on the concept of PAINS (pan-assay interference compounds), compounds that have apparent activity in multiple assays, but in fact produce false signals due to non-specific interactions or interference with the assays. As indicated by Baell and Walters (2014) in a recent commentary, PAINS can lead to confusion and wasted resources. In this paper, Bajorath points out that promiscuity, one of the characteristics of PAINS, can also be due to the ability of small molecules to specifically interact with multiple targets, which provides the molecular basis of ‘polypharmacology.'  This paper highlights the difficulties that may be encountered in distinguishing between these two possibilities.

I have read this submission. I believe that I have an appropriate level of expertise to confirm that it is of an acceptable scientific standard.

# Peer review: Referee response for version 1

**DOI:** 10.5256/f1000research.5795.r6317

**Published:** 2014-10-06

**Authors:** John Lowe

**Affiliations:** 1 JL3Pharma LLC, Stonington, USA

The authors make a good case for the importance of the recent publication on PAINS compounds, hits from screening that are artifacts and may drain resources in followup form legitimate hits.  Their conclusion that it will take years for the scientific community to accept this case and institute controls to protect against it is also valid.  They point some caveats in terms of compounds that are legitimately promiscuous and therapeutically valuable as a result.  But for academic labs unfamiliar with the HTS triage process, the danger of wasting resource on PAINS will be an ongoing risk of the drug discovery process.  The article thus makes many valid and worthwhile points, and should be indexed.

I have read this submission. I believe that I have an appropriate level of expertise to confirm that it is of an acceptable scientific standard.
